# Electromyographic respiratory muscle analysis in the spontaneous breathing trial: a randomized crossover trial comparing T-piece and pressure support

**DOI:** 10.62675/2965-2774.20260486

**Published:** 2026-08-21

**Authors:** Diogo Fanfa Bordin, Robledo Leal Condessa, Éder Kroeff Cardoso, Graciele Sbruzzi, Bruna Ziegler

**Affiliations:** 1 Universidade Federal do Rio Grande do Sul Faculdade de Medicina Postraduate Program in Pulmonology Porto Alegre RS Brazil Postraduate Program in Pulmonology, Faculdade de Medicina, Universidade Federal do Rio Grande do Sul - Porto Alegre (RS), Brazil.; 2 Universidade Federal do Rio Grande do Sul Hospital de Clínicas de Porto Alegre Physiotherapy Service Porto Alegre RS Brazil Physiotherapy Service, Hospital de Clínicas de Porto Alegre, Universidade Federal do Rio Grande do Sul - Porto Alegre (RS), Brazil.; 3 Hospital de Pronto Socorro de Porto Alegre Physiotherapy Service Porto Alegre RS Brazil Physiotherapy Service, Hospital de Pronto Socorro de Porto Alegre - Porto Alegre (RS), Brazil.

**Keywords:** Ventilatory weaning, Electromyography, Respiratory muscles, Physical therapy

## Abstract

**Objective:**

To compare the electromyographic activity of respiratory muscles during T-piece and pressure support ventilation trials.

**Methods:**

This was a randomized crossover trial involving 16 patients weaning from mechanical ventilation. Each patient underwent two 30-minute spontaneous breathing trials (T-piece and pressure-support ventilation with 7cmH_2_O), separated by a 30-minute washout period, or until vital signs returned to baseline. Surface electromyography was used to assess muscle activation (percentage root mean square) and median frequency of the pectoralis major, rectus abdominis, and external intercostal muscles (nomenclature updated from diaphragm based on SENIAM and Phinyomark/De Troyer literature to account for crosstalk). Data were collected at baseline (T0), 15 (T15), and 30 minutes (T30). Intragroup comparisons were performed using two-way repeated-measures ANOVA, and intergroup differences in deltas (Δ) were analyzed using ANCOVA, adjusting for baseline values.

**Results:**

Median frequency remained stable for all muscles (p > 0.05), indicating no electromyographic fatigue. In contrast, percentage root mean square activation differed significantly between methods. During the T-piece spontaneous breathing trial, a progressive increase in percentage root mean square was observed in the pectoralis major, rectus abdominis, and external intercostals (p < 0.05). Under pressure support ventilation, only the pectoralis major showed a modest increase (p < 0.05), while other muscles remained stable. Intergroup comparisons of pre-to-post variation confirmed greater activation during the T-piece spontaneous breathing trial for the pectoralis major (p = 0.048) and rectus abdominis (p = 0.009). No significant differences were observed between methods for the external intercostal muscles.

**Conclusions:**

T-piece ventilation induces greater recruitment of thoracic and abdominal accessory respiratory muscles than pressure-support ventilation, reflecting a higher ventilatory demand. Stable median frequency values indicate preserved neuromuscular function and absence of clinically relevant respiratory muscle fatigue over 30 minutes. These findings suggest that pressure support ventilation partially unloads the respiratory system and may mask true ventilatory effort, whereas the T-piece more closely reproduces post-extubation conditions. Surface electromyography proved to be a feasible tool for physiological assessment during ventilatory weaning.

## INTRODUCTION

Weaning from mechanical ventilation (MV) is defined as the process of gradually reducing ventilatory support and accounts for more than 40% of the total duration of MV,^(1)^ and this proportion may be even higher depending on the etiology of respiratory failure.^(1-4)^ Among the factors associated with extubation failure are ventilator-associated pneumonia,^(5)^ delirium,^(6)^ diaphragmatic dysfunction,^(7)^ critical illness polyneuropathy,^(8)^ and laryngeal stridor.^(3,4)^ Despite the implementation of standardized protocols and the accumulated experience of several intensive care units (ICUs), extubation failure still occurs in approximately 20% of cases.^(2)^ In order to improve weaning outcomes and minimize extubation failure, several tests have been recommended to predict and identify the optimal timing for extubation.^(3,4)^ According to Rothaar et al., patients who experience extubation failure present mortality rates 2.5 to 10 times higher than those successfully extubated.^(9)^

The spontaneous breathing trial (SBT) is a simple and effective tool for assessing the respiratory system's ability to sustain spontaneous ventilation prior to extubation. It can be performed using a T-piece (T-tube [TT]) or low-level pressure-support ventilation (PSV), usually up to 7cmH_2_O.^(3,4)^ To date, no clear superiority has been demonstrated between these two methods (T-piece *versus* PSV) regarding major clinical outcomes,^(10)^ except SBT success, which appears to favor PSV. Nevertheless, physiological evidence indicates that different SBT techniques impose markedly different workloads on the respiratory muscles. A physiologic meta-analysis by Sklar et al. demonstrated that PSV significantly reduces inspiratory effort compared to the T-piece, which more closely mimics the work of breathing observed after extubation.^(11)^

Respiratory muscle fatigue is one of the main factors associated with extubation failure. Therefore, it is recommended that patients remain on spontaneous breathing for at least 30 minutes without hemodynamic changes greater than 10% from baseline at the start of the test.^(12)^ In the literature, some studies^(13,14)^ have used surface electromyography (EMG) to analyze respiratory muscle activation^(15)^ and muscle fatigue,^(16,17)^ particularly in difficult weaning of tracheostomized patients^(18)^ and in predicting weaning outcomes after cardiovascular surgery.^(19)^ Surface EMG allows the identification of action potentials generated by motor units of muscle fibers and thus provides detailed information regarding muscle behavior.^(16,20,21)^

However, few studies^(15,18,19)^ have evaluated the use of EMG in the ICU setting and during the MV weaning process. Moreover, to date, no studies have investigated this outcome comparing T-piece *versus* PSV during the SBT. Therefore, this study aims to evaluate respiratory muscle behavior using surface EMG during SBTs performed with a T-piece versus pressure-support ventilation.

## METHODS

### Trial design

This is a randomized crossover clinical trial conducted in a single adult ICU. The study aimed to evaluate the EMG activity of respiratory muscles during two standardized SBT methods: T-piece (intervention A) and PSV (intervention B). The sequence of SBT methods was implemented using a balanced sequential order (1:1 allocation) to control for potential order effects. Each participant served as their own control. A minimum washout period of 30 minutes was observed between trials, or until vital signs - including heart rate (HR), respiratory rate (RR), blood pressure (BP), and peripheral oxygen saturation (SpO_2_) - returned to baseline values, as previously proposed by dos Santos et al.^(21)^ The study followed the CONSORT 2010 statement extension for randomized crossover trials.

### Participants and eligibility

Inclusion criteria was age ≥ 18 years, invasive MV for at least 48 hours, and readiness for ventilatory weaning according to institutional protocols based on the Brazilian Recommendations for Mechanical Ventilation,^(3,4)^ defined as resolution or clear improvement of the cause of acute respiratory failure, adequate level of consciousness with the ability to initiate spontaneous respiratory effort, hemodynamic stability without uncontrolled arrhythmias and without vasopressor support or requiring only low and stable doses, adequate gas exchange (peripheral oxygen saturation ≥ 90% with fraction of inspired oxygen ≤ 0.40 and positive end-expiratory pressure [PEEP] ≤ 8cmH_2_O), and absence of significant respiratory acidosis or progressive acid-base imbalance. Exclusion criteria included previous weaning failure, chronic obstructive pulmonary disease, hemiparesis or hemiplegia, delirium and/or psychomotor agitation, tracheostomy, recent postoperative status following cardiothoracic surgery with sternotomy or abdominal surgery with laparotomy that precluded electrode placement, as well as refusal of informed consent by family members.

### Setting and period

This Ethics Committee-approved study was conducted at the *Hospital de Clínicas de Porto Alegre* adult ICU (March - July 2018) and registered as NCT07387731 (ClinicalTrials.gov).

### Interventions

Before starting the SBT, all patients underwent airway suctioning per institutional protocol to ensure airway patency. Additionally, patients were suctioned whenever coughing or hypersecretion occurred during the trials to prevent failure due to excessive secretions, as suctioning is recommended and performed as needed to maintain clinical stability. During the T-piece trial, supplemental oxygen was delivered at 1 to 5L/min. Tidal volume (TV) during this trial was measured using an analog Wright^®^ respirometer (Mark 8 model) connected to the T-piece. During the PSV trial, pressure support was set at 7cmH_2_O with PEEP between 5 and 6cmH_2_O, maintaining baseline O_2_. Success in the SBT was defined as the absence of signs of intolerance (RR > 35bpm, SpO_2_ < 90%, HR > 140bpm, SBP > 180mmHg or < 90mmHg, and signs of increased work of breathing) after 30 minutes, in accordance with the Brazilian Recommendations for MV.^(3,4)^ Extubation failure was defined as the need for reintubation within 48 hours after planned extubation.

### Outcomes

The primary outcomes were the electromyographic variables: median frequency (MF) and percentage root mean square (%RMS) of the pectoralis major, rectus abdominis, and external intercostal muscles at the fifth intercostal space along the anterior axillary line (right and left sides). Secondary outcomes included hemodynamic variables (HR, BP), ventilatory parameters (RR, VT, SpO_2_), and the frequency of airway suctioning, reintubation within 48 hours, ICU mortality and readmission within 30 days, and ICU length of stay. Measurements were recorded at baseline, 15, and 30 minutes.

### Electromyography assessment

Surface EMG (sEMG) followed the Surface ElectroMyoGraphy for the Non-Invasive Assessment of Muscles recommendations (SENIAM).^(22)^ Initially, electrodes placed at the fifth intercostal space along the anterior axillary line were intended to capture diaphragmatic activity. However, given the significant risk of crosstalk,^(23)^ and the established inspiratory mechanical advantage of the external intercostals in the lower rib cage as described by De Troyer et al.,^(24)^ the nomenclature was updated to "external intercostal muscles" to ensure methodological accuracy. Skin was prepared with trichotomy and cleansing to reduce impedance.^(22-25)^ A surface electromyograph (MIOTEC, Miotool 400, Brazil) with a 2,000 Hz sampling frequency was used. To ensure that EMG signals corresponded specifically to the inspiratory phase, a webcam was used to synchronize the ventilatory pattern with the EMG tracings. Data extraction was performed by a researcher blinded to the SBT sequence.

### Sample size

Based on a pilot study of five individuals, a 20% variation in the %RMS of the rectus abdominis was considered clinically relevant. With a 5% significance level and 80% power, the minimum sample size was estimated at 16 patients.

### Randomization and blinding

Trials were randomized (1:1) by an independent researcher using the Bracket RTSM application. While the care team remained unblinded to the SBT method, the researcher analyzing EMG signals was blinded to the sequence.

### Statistical analysis

Data were analyzed using SPSS 18.0 and Stata 11.2, with normality confirmed by the Shapiro-Wilk test. A two-way repeated measures analysis of variance (ANOVA) assessed method, period, carry-over, and intragroup effects. Electromyography variables (%RMS and MF) were compared via analysis of covariance (ANCOVA), adjusted for baseline. To prevent crossover bias, only patients completing both SBTs were included (per-protocol). Significance was set at 5% (two-tailed).

## RESULTS

**Figure 1:**
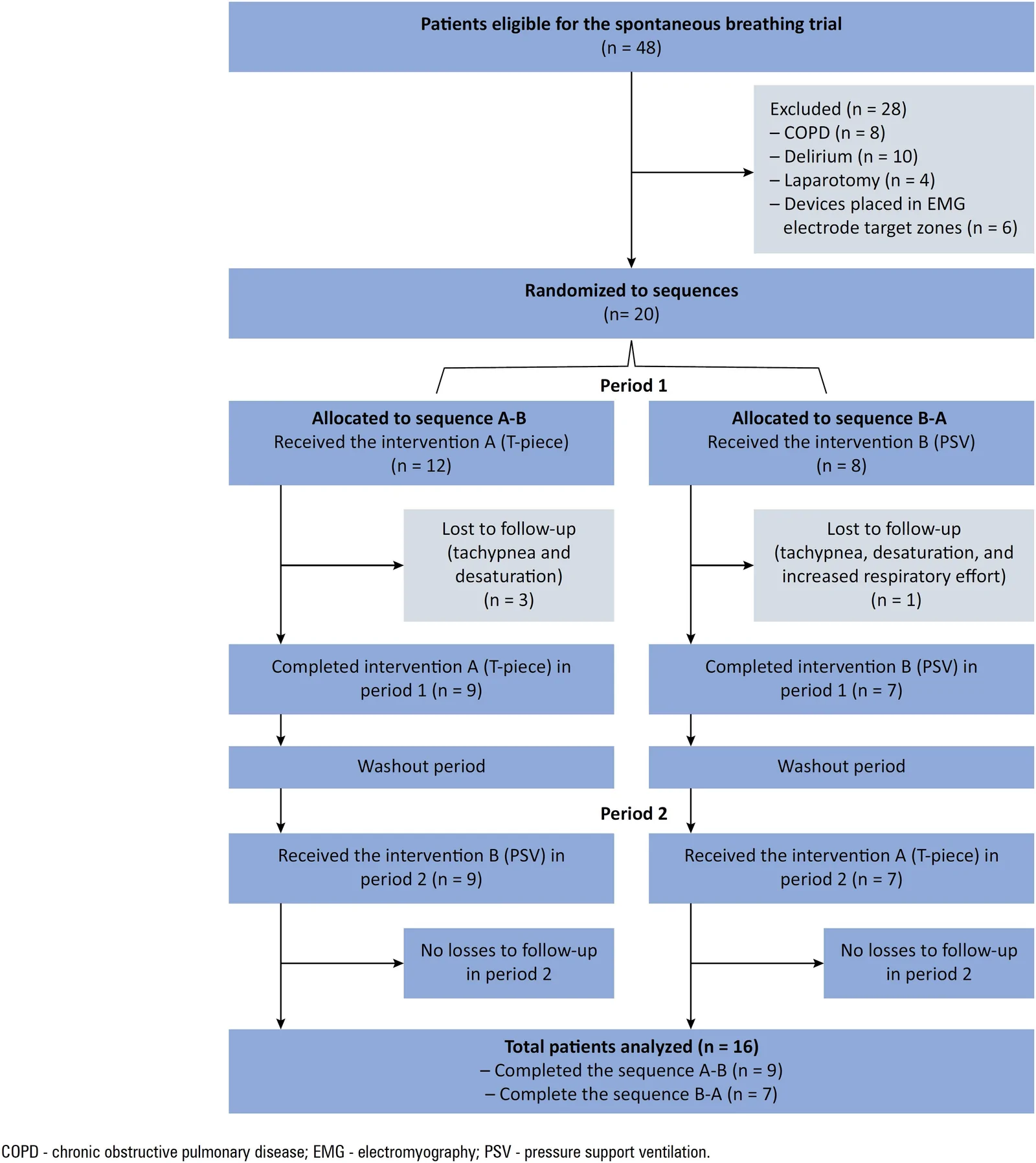
Patients included in the study.

During the study period, 48 patients were identified as eligible to undergo an SBT. Of these, 20 patients were selected to undergo testing, and 16 were ultimately analyzed in the study using a crossover design for both the PSV and T-piece methods (Figure 1). Four patients were excluded due to premature SBT failure caused by severe psychomotor agitation; resulting movement artifacts compromised the reliability of electromyographic data for interpretation. All excluded patients failed their first SBT, with three failing the T-piece trial and one the PSV trial. All randomized patients included in the final analysis (n = 16) achieved successful ventilatory weaning.

Nine patients (56.3%) required orotracheal intubation for acute respiratory failure, while the remaining seven (43.7%) had other clinical causes (Table 1). In the carryover effect analysis of baseline values for all studied variables, a carryover effect was observed only for MF in the rectus abdominis muscle (p = 0.039). No carryover effect was identified for the other study variables; this effect was corrected using ANCOVA. The need for airway suctioning between methods was also evaluated. The PSV method presented a median of zero (zero to one), whereas the T-piece presented a median of 1 (zero to three) (p = 0.007). A statistically significant interaction was found between the number of suctioning events and the electromyographic variations between periods. Regarding secondary outcomes, no cases of reintubation within 48 hours or ICU mortality within 30 days were observed [zero (0%)]. The 30-day ICU readmission rate was 12.5% (n = 2). The median ICU length of stay was 3 (1 - 4) days.

**Table 1:** General characteristics of the studied patients

Characteristics			Total (n = 16)
Age (years)			56.25 ± 13.78
Gender			
	Male		8 (50)
	Female		8 (50)
APACHE II (score)			20.3 ± 9.7
Time on mechanical ventilation (hours)			47.94 ± 13.47
Causes of intubation			
	Acute respiratory failure		9 (56.3)
		Pneumocystosis	2 (12.8)
		Aspirative pneumonia	2 (12.8)
		Community-acquired pneumonia	1 (6.3)
		Respiratory infection	1 (6.3)
		Acute cardiogenic pulmonary edema	3 (18.5)
	Other causes		7 (43.7)
		Convulsive crisis	2 (12.8)
		Asthma	2 (12.8)
		Diabetic ketoacidosis	1 (6.3)
		Decreased level of consciousness	1 (6.3)
Mechanical ventilation parameters			
	PEEP (cmH_2_O)		6 ± 0.6
	PS-PEEP (cmH_2_O)		11.31 ± 1.4
	FiO_2_ (%)		30.13 ± 4.9
	TV (mL)		520.63 ± 89.62

APACHE - Acute Physiology and Chronic Health Evaluation; PEEP - positive end-expiratory pressure; PS-PEEP - pressure support above positive end-expiratory pressure; FiO_2_ - fraction of inspired oxygen; TV - tidal volume. Results expressed as mean ± standard deviation of n (%).

**Figure 2:**
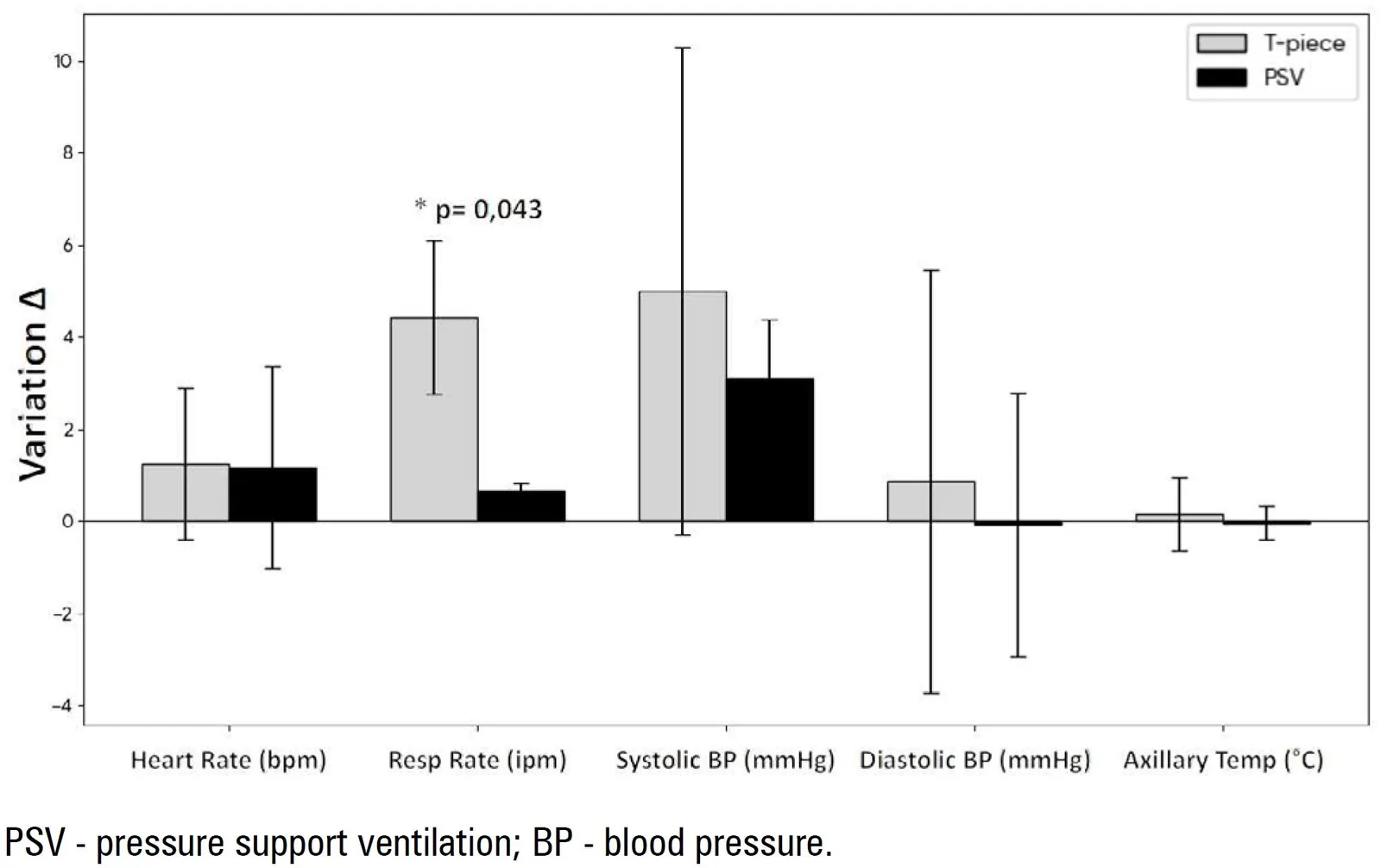
Comparison of the variation Δ in hemodynamic parameters and axillary temperature between T-piece and pressure support ventilation weaning methods.

**Figure 3:**
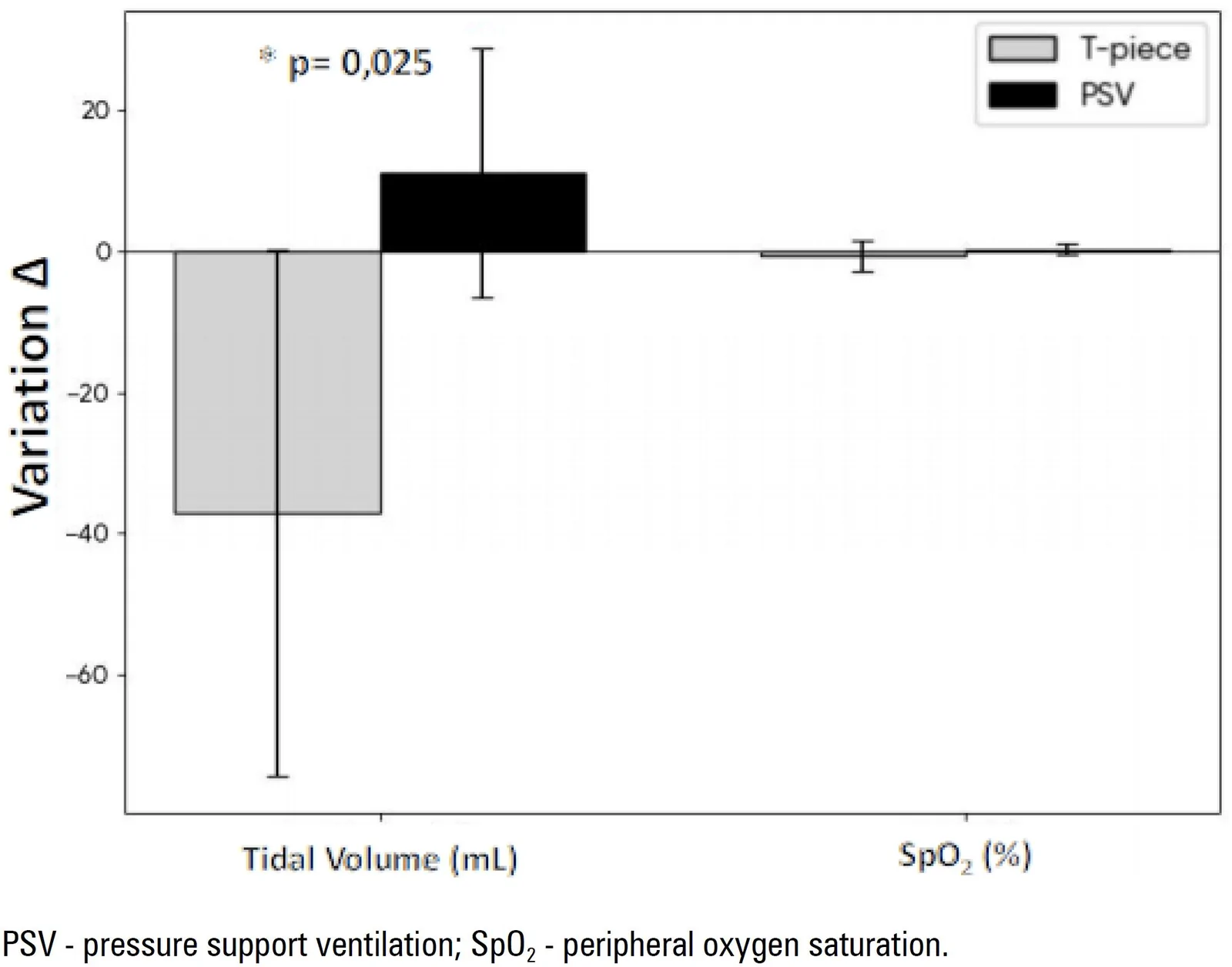
Comparison of the variation Δ in tidal volume and peripheral oxygen saturation between T-piece and pressure support ventilation weaning methods.

No statistically significant differences were observed between the SBT methods regarding changes (Δ) in hemodynamic and oxygenation variables (Figure 2). In contrast, statistically significant differences were observed for RR and VT (Figure 3), with a greater increase during the T-piece SBT (4.43 ± 1.67 breaths/minute) compared to PSV (0.65 ± 0.18 breaths/minute; p = 0.043), as well as for a VT, which decreased during the T-piece SBT (-37.18 ± 37.23mL) and increased during PSV (11.00 ± 17.70mL; p = 0.025). Absolute values and ANCOVA-derived delta comparisons with 95% confidence intervals are detailed in table 1S (Supplementary Material).

**Figure 4:**
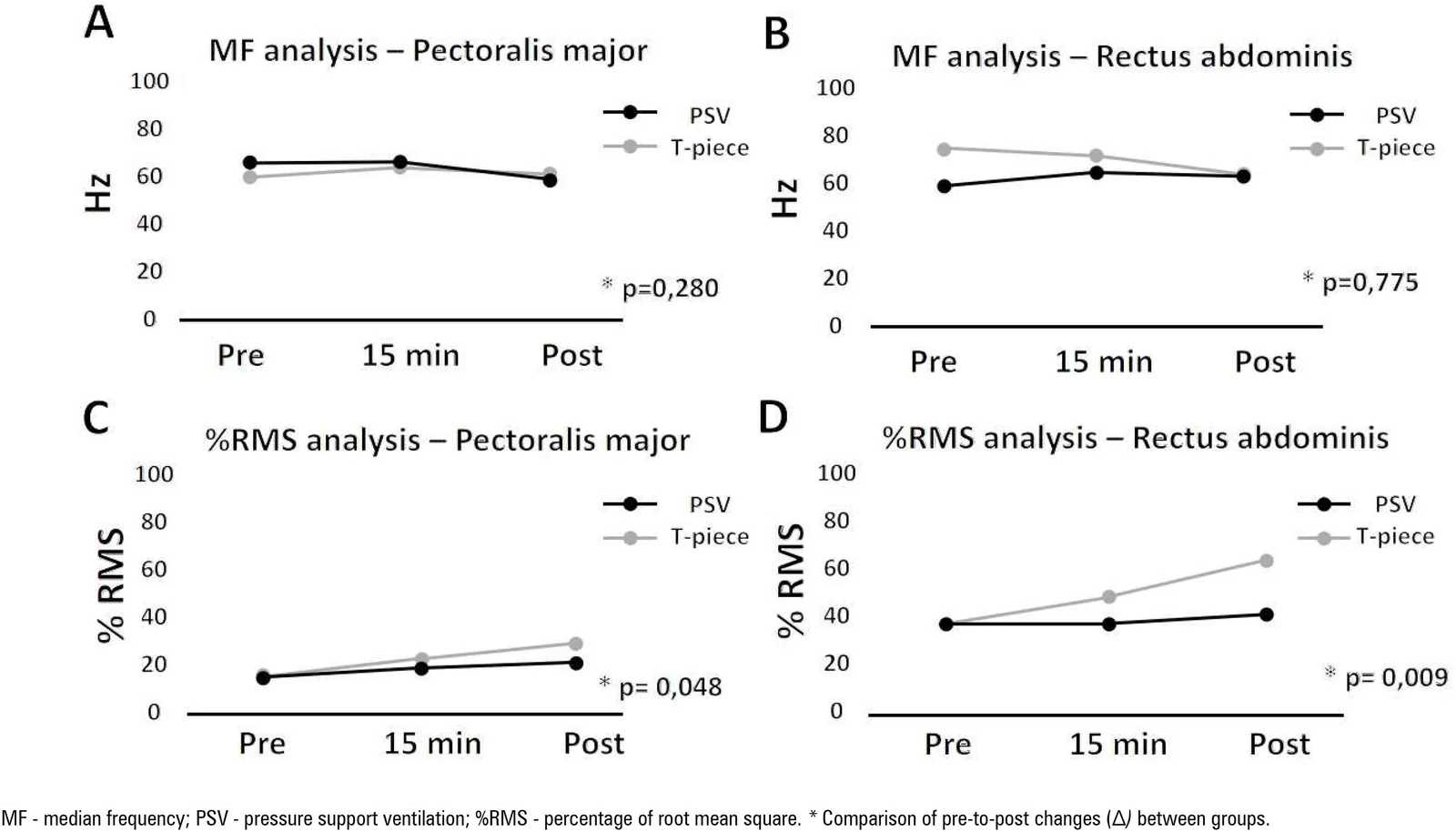
Intragroup analysis of the percentage of root mean square and median frequency in the pectoralis major and rectus abdominis muscles.

**Figure 5:**
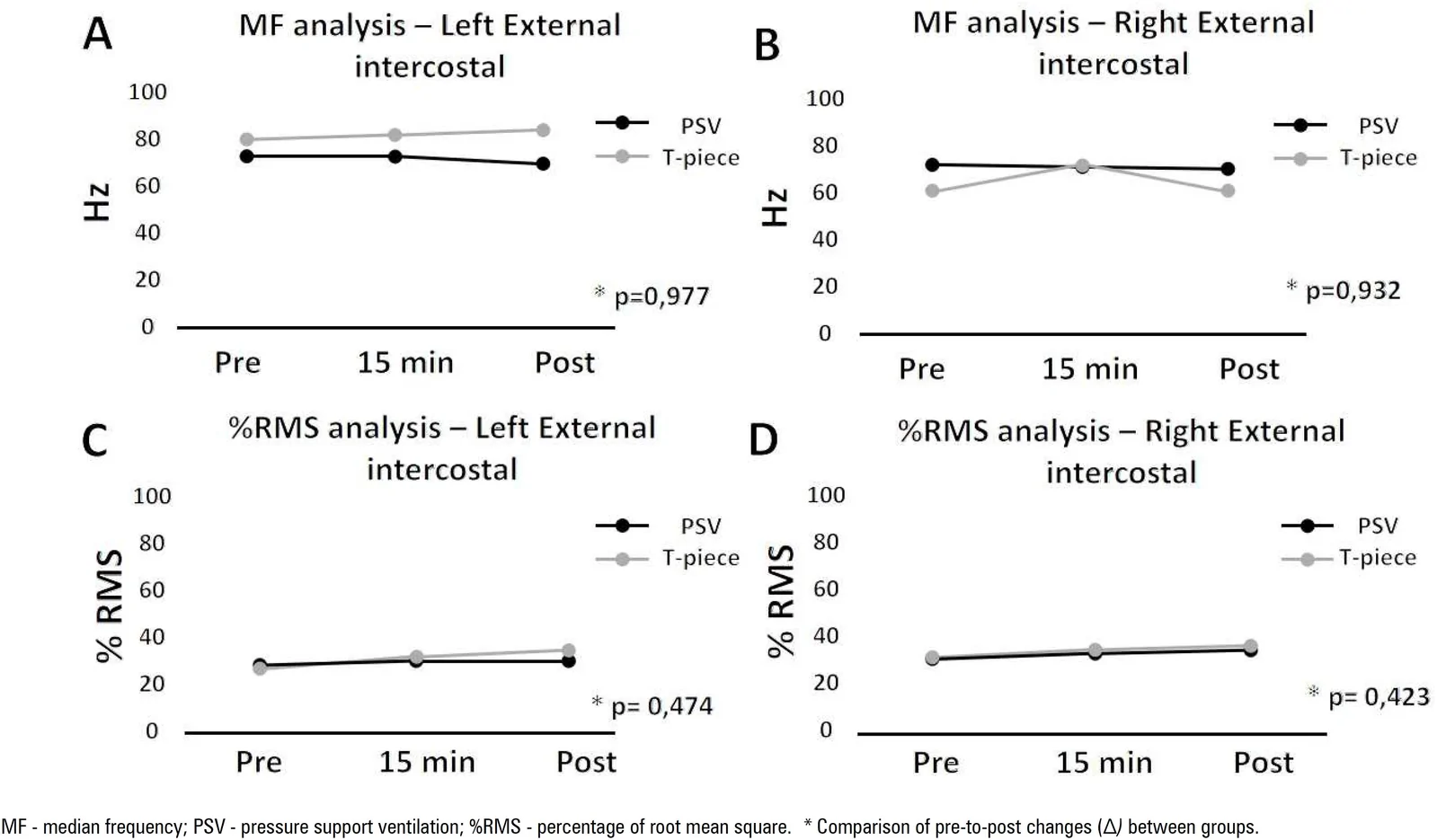
Intragroup analysis of the percentage of root mean square and median frequency in the external intercostal muscles.

Figures 4 and 5 illustrate the electromyographic responses. While MF remained stable across all muscles, muscle activation (%RMS) increased significantly and progressively during the T-piece SBT compared to PSV. Regarding the comparison of pre-to-post changes (Δ) between groups, significant differences were observed for the pectoralis major (p = 0.048) and rectus abdominis (p = 0.009). In contrast, no significant intergroup differences were found for the external intercostals (left: p = 0.474; right: p = 0.423). Detailed absolute values, along with ANOVA and ANCOVA comparisons, are provided in tables 2S and 3S (Supplementary Material).

ANCOVA test, statistical significance set at p = 0.05.

ANCOVA test, statistical significance set at p = 0.05.

## DISCUSSION

In the present study, we evaluated 16 patients during weaning from invasive MV, all of whom completed the SBT and subsequent extubation. Compared with PSV, the T-piece SBT was associated with greater electromyographic activation, expressed by increased %RMS in the pectoralis major and rectus abdominis. In contrast, no between-method differences were observed in MF. The primary outcome was the comparison of %RMS variation between SBT methods in these muscles. These findings align with physiological evidence showing that different SBT techniques impose distinct respiratory muscle workloads. Pressure support-based SBTs partially unload inspiratory muscles by reducing elastic and resistive loads, whereas T-piece trials more closely resemble post-extubation conditions. Consequently, T-piece SBT promotes greater accessory muscle recruitment, reflecting higher ventilatory demand rather than pathological overload, as described in studies assessing work of breathing, pressure-time product, and respiratory muscle activation during weaning.^(11,26-28)^

Although electromyographic evaluation during weaning has traditionally focused on the sternocleidomastoid due to its role as a major accessory inspiratory muscle,^(2,15,25)^ more recent physiological investigations highlight thoracic and abdominal accessory muscles as indirect markers of ventilatory cost during spontaneous breathing.^(26,29.30)^ In this context, selecting the pectoralis major and rectus abdominis is appropriate, given their roles in chest wall stabilization and expiratory assistance, while minimizing interference from cervical vascular devices and improving signal reliability.

Moreover, reporting external intercostal muscle activity instead of diaphragmatic activity represents a methodological refinement based on greater anatomical accuracy. Although the fifth intercostal space is commonly associated with the diaphragmatic zone of apposition, surface EMG at this level is susceptible to crosstalk from more superficial muscles,^(23)^ such as the external intercostals, which play an important inspiratory mechanical role and are predictably recruited to assist the diaphragm during increased ventilatory work.^(24)^ Thus, the progressive increase in %RMS observed during the T-piece trial reflects greater recruitment of accessory muscles to sustain ventilation under higher resistive loads.

### Respiratory mechanics and loss of physiological positive end-expiratory pressure during T-piece spontaneous breathing trial

A relevant physiological mechanism underlying the greater electromyographic activation observed during the T-piece SBT is the loss of physiological positive PEEP. During PSV, the application of external PEEP helps maintain functional residual capacity (FRC), improves lung compliance, and reduces the elastic workload on the respiratory system.^(11,29)^ In contrast, during the T-piece SBT, the abrupt withdrawal of both pressure support and PEEP leads to a reduction in end-expiratory lung volume, promoting partial alveolar derecruitment and a decrease in FRC.^(26,31)^

This reduction in FRC shifts tidal breathing to lower lung volumes, increasing elastic recoil and neuromechanical dissociation. As a compensatory response, accessory inspiratory and expiratory muscles are recruited to stabilize the thoracoabdominal compartment and maintain ventilation. Physiological studies have shown that lower lung volumes and higher elastic loads are associated with increased recruitment of the chest wall and abdominal muscles.^(26,31)^ Therefore, the greater activation of the pectoralis major and rectus abdominis during the T-piece SBT likely reflects an adaptive response to increased ventilatory demand due to loss of physiological PEEP rather than an isolated increase in respiratory drive.

### Ventilator settings and potential masking of respiratory effort during pressure support ventilation

Another important factor relates to the ventilator settings used during the PSV-SBT. In the present study, PSV was conducted with a pressure support level of 7cmH_2_O and a PEEP of 5cmH_2_O, which is consistent with international guidelines and routine clinical practice. However, physiological studies consistently demonstrate that even low levels of pressure support significantly unload the respiratory muscles by reducing inspiratory effort, esophageal pressure swings, and diaphragmatic work.^(11,28.29)^

This ventilatory assistance may partially mask true respiratory workload, particularly in patients with limited reserve. By reducing both resistive and elastic components of the work of breathing, PSV decreases accessory muscle recruitment, consistent with the lower %RMS observed in the pectoralis major and rectus abdominis during the SBT using PSV. Randomized trials and network meta-analyses have shown that SBT performed with PSV is associated with lower physiological effort than T-piece trials, which more closely replicate post-extubation mechanics.^(27,32)^ Thus, the reduced electromyographic activation observed during PSV likely reflects ventilatory unloading rather than superior neuromuscular efficiency.

### Muscle recruitment versus fatigue: interpretation of %RMS and median frequency findings

A key finding of the present study is the dissociation between increased muscle recruitment and the absence of electromyographic fatigue. The significant increase in %RMS during the SBT using T-piece, without a concomitant reduction in MF, suggests that this method imposes greater respiratory muscle workload but does not necessarily induce muscle fatigue within the 30-minute evaluation period.^(33)^

Median frequency is widely recognized as a sensitive parameter for detecting changes in muscle fiber conduction velocity and spectral compression toward lower frequencies, which are hallmarks of peripheral muscle fatigue.^(16,25)^ More recent evidence indicates that the absence of MF reduction during submaximal efforts reflects preserved contractile capacity and effective neuromuscular adaptation.^(34)^ In this context, the stable MF values observed in our cohort suggest that, although the SBT performed with a T-piece requires increased muscle recruitment, the imposed load remains within a tolerable physiological range for patients who ultimately succeed in weaning.

This interpretation is supported by recent studies evaluating the electrical activity of the diaphragm during SBTs, which demonstrate that increased neural drive and muscle activation are common with more demanding weaning strategies. However, fatigue-related spectral changes are typically observed in patients who fail the SBT rather than in those who complete it.^(18,30)^ Therefore, the increased %RMS without MF decline observed in the present study is consistent with a state of elevated ventilatory demand rather than impending respiratory muscle exhaustion.

### Clinical variables and ventilatory demand

The greater increase in RR observed during the SBT performed with T-piece is consistent with recent systematic reviews and meta-analyses comparing T-piece and PSV strategies, which report higher RR and lower TVs with the T-piece method.^(10,27)^ These ventilatory adjustments reflect compensatory mechanisms to overcome the increased elastic and resistive loads imposed by the absence of ventilatory assistance and the presence of the endotracheal tube.^(26,35)^

Although airway suctioning was more frequent during the SBT using a T-piece, no significant association was observed between suctioning events and electromyographic variables. This finding suggests that the increased muscle activation is primarily related to sustained ventilatory mechanics rather than transient events such as coughing.

### Limitations

While our findings provide important insights, certain limitations warrant consideration. The single-center design and small sample size may affect generalizability. Regarding the crossover design, a carry-over effect detected for rectus abdominis MF was addressed via ANCOVA, though residual neuromuscular adaptation cannot be fully excluded. Nevertheless, this work underscores surface EMG as a feasible, non-invasive physiological tool. By clarifying muscle recruitment and ventilatory cost, surface EMG enhances the understanding of weaning physiology, supporting individualized strategies in complex clinical scenarios.^(36)^

Finally, technical challenges related to isolating deep respiratory muscles with surface EMG must be considered. To minimize anatomical misinterpretation due to signal crosstalk, the nomenclature was updated from "diaphragm" to "external intercostal muscles", in accordance with SENIAM recommendations and physiological descriptions indicating predominance of superficial muscle signals at the fifth intercostal space.^(24)^

## CONCLUSION

This study demonstrated that spontaneous breathing trials performed with a T-piece are associated with greater electromyographic activation of accessory respiratory muscles, particularly the pectoralis major and rectus abdominis, compared with pressure support ventilation. This increased muscle recruitment occurred alongside a higher respiratory rate and reduced tidal volume, indicating greater ventilatory demand during the spontaneous breathing trial under the T-piece. Importantly, despite the higher root mean square observed with the T-piece, median frequency remained stable across all muscles, suggesting preserved neuromuscular function and the absence of clinically relevant respiratory muscle fatigue during the 30-minute evaluation period.

These findings indicate that the spontaneous breathing trial performed with a T-piece imposes a higher physiological load, likely related to the withdrawal of pressure support and positive end-expiratory pressure. In contrast, pressure support ventilation partially unloads the respiratory system and may mask true ventilatory effort. Surface electromyography proved to be a feasible and informative tool for assessing respiratory muscle recruitment during weaning. Future multicenter studies with larger sample sizes are needed to further define the role of surface electromyography as a complementary physiological tool for assessing ventilatory weaning.

## Data Availability

The data underlying this manuscript are not publicly available due to ethical and confidentiality restrictions regarding patient information. However, anonymized data may be made available from the corresponding author upon reasonable request and subject to approval by the appropriate ethics committee. The methods, procedures, and statistical analyses are fully described in the manuscript.
