# Supplementary material for: Electromyographic respiratory muscle analysis in the spontaneous breathing trial: a randomized crossover trial comparing T-piece and pressure support
Source: Crit Care Sci. 2026 Aug 21;38:e20260486. doi: 10.62675/2965-2774.20260486 (PMC13518006; doi:10.62675/2965-2774.20260486)
Supplement: Supplementary file 1 [file 2965-2774-ccsci-38-e20260486-Supp01.pdf]

Critical Care Science
https://doi.org/10.62675/2965-2774.20260486

SUPPLEMENTARY MATERIAL

Electromyographic respiratory muscle analysis in the spontaneous breathing trial: a randomized crossover trial comparing T-piece and pressure support

Diogo Fanfa Bordin[1], Robledo Leal Condessa[2], Éder Kroeff Cardoso[3], Graciele Sbruzzi[1], Bruna Ziegler[1]

Table 1S - Comparison of hemodynamic, oxygenation, and ventilatory changes (Δ) during spontaneous breathing trial methods

Variables	T-piece (n = 16)	PSV (n = 16)	p value
Hemodynamics and oxygenation			
Δ HR (bpm)	1.25 ± 1.64	1.18 ± 2.19	0,918
Δ SBP (mmHg)	5.00 ± 5.30	3.12 ± 1.25	0,184
Δ DBP (mmHg)	0.87 ± 4.58	-0.06 ± 2.86	0,492
Δ AT (°C)	0.16 ± 0.79	-0.03 ± 0.36	0,385
Δ SpO_2 (%)	-0.75 ± 2.23	0.18 ± 0.75	0,126
Ventilatory parameters			
Δ RR (breaths/min)	4.43 ± 1.67	0.65 ± 0.18	0.043
Δ VT (mL)	-37.18 ± 37.23	11.00 ± 17.70	0.025

PSV - pressure support ventilation; HR - heart rate; SBP - systolic blood pressure; DBP - diastolic blood pressure; AT - axillary temperature; SpO_2 - peripheral oxygen saturation; RR - respiratory rate; VT - tidal volume. $p > 0.05$. Results are expressed as mean ± standard deviation.

Table 2S - Analysis of Δ electromyographic variables between groups

Variables	T-piece	PSV	Adjusted difference (95%CI)	p value
MF (Hz)				
Pectoralis major	1.44 ± 19.48	-5.91 ± 22.04	7.35 (-7.24 to 21.94)	0.280
Rectus abdominis	-10.97 ± 23.94	4.54 ± 13.71	-15.51 (-29.35 to -1.67)	0.775
Left external intercostal	5.58 ± 23.73	-2.78 ± 20.91	8.36 (-7.35 to 24.07)	0.977
Right external intercostal	1.69 ± 15.44	-0.05 ± 20.92	1.74 (-11.12 to 14.60)	0.932
Root mean square (%)				
Pectoralis major	10.25 ± 7.49	5.72 ± 4.98	4.53 (0.05 to 9.01)	0.048*
Rectus abdominis	26.18 ± 12.78	3.92 ± 11.76	22.26 (13.63 to 30.89)	0.009*
Left external intercostal	9.47 ± 10.32	3.86 ± 6.47	5.61 (-0.46 to 11.68)	0.474
Right external intercostal	6.72 ± 6.60	5.28 ± 4.49	1.44 (-2.48 to 5.36)	0.423

PSV - pressure support ventilation; 95%CI - 95% confidence interval; MF - median frequency. * ANCOVA test, statistical significance at $p < 0.05$. Values are expressed as mean ± standard deviation for electromyographic variables between the T-piece and pressure-support ventilation groups, including the adjusted difference and 95% confidence interval.

This is an open access article under the CC BY license (https://creativecommons.org/licenses/by/4.0/).

Table 3S - Absolute values and intragroup analysis (ANOVA) of electromyographic variables

Muscle/variable	Group	Pre (T0)	15 min (T15)	Post (T30)	p1 (T0 - T15)	p2 (T15 - T30)	p3 (T0 - T30)
Pectoralis major							
MF (Hz)	PSV	66.0 ± 8.2	66.5 ± 7.5	59.0 ± 9.1	0.880	0.210	0.150
	T-piece	60.0 ± 9.5	64.0 ± 8.8	62.0 ± 10.2	0.450	0.720	0.610
%RMS	PSV	15.0 ± 3.1	19.0 ± 4.2	21.0 ± 5.0	0.120	0.350	0.045
	T-piece	15.0 ± 3.5	23.0 ± 6.1	30.0 ± 7.4	0.038	0.041	< 0.001
Rectus abdominis							
MF (Hz)	PSV	59.0 ± 11.2	64.0 ± 10.5	63.0 ± 12.1	0.520	0.890	0.640
	T-piece	74.0 ± 13.4	72.0 ± 12.8	63.0 ± 14.2	0.710	0.180	0.090
%RMS	PSV	37.0 ± 9.2	37.5 ± 8.8	41.0 ± 10.4	0.910	0.480	0.220
	T-piece	37.0 ± 10.5	48.0 ± 12.1	64.0 ± 15.6	0.015	0.008	< 0.001

T0 - pre-spontaneous breathing trial baseline; T15 - 15 minutes of trial; T30 - 30 minutes (post-spontaneous breathing trial); MF - median frequency; PSV - pressure support; %RMS - percentage of root mean square; PSV - pressure support ventilation. Intragroup Analysis (ANOVA): p1 compares T0 *versus* T15; p2 compares T15 *versus* T30; p3 compares T0 *versus* T30. A p-value < 0.05 denotes a statistically significant difference in the pairwise longitudinal comparison using the Bonferroni post-hoc test. The p values displayed in figure 4 refer to the intergroup ANCOVA analysis, adjusting for baseline values. Data are expressed as mean ± standard deviation for absolute values.
